# The palladacycle complex AJ-5 induces apoptotic cell death while reducing autophagic flux in rhabdomyosarcoma cells

**DOI:** 10.1038/s41420-019-0139-9

**Published:** 2019-01-28

**Authors:** Jenna Susan Bleloch, André du Toit, Liezl Gibhard, Serah Kimani, Reyna Deeya Ballim, Minkyu Lee, Angelique Blanckenberg, Selwyn Mapolie, Lubbe Wiesner, Ben Loos, Sharon Prince

**Affiliations:** 1 Division of Cell Biology, Department of Human Biology, Faculty of Health Sciences, University of Cape Town, Cape Town, Western Cape South Africa; 2 Department of Physiological Sciences, Stellenbosch University, Stellenbosch, Western Cape South Africa; 3 Division of Clinical Pharmacology, Department of Medicine, Faculty of Health Sciences, University of Cape Town, Cape Town, Western Cape South Africa; 4 Department of Chemistry and Polymer Science, Stellenbosch University, Stellenbosch, Western Cape South Africa

## Abstract

Rhabdomyosarcoma (RMS) forms in skeletal muscle and is the most common soft tissue sarcoma in children and adolescents. Current treatment is associated with debilitating side effects and treatment outcomes for patients with metastatic disease are dismal. Recently, a novel binuclear palladacycle, AJ-5, was shown to exert potent cytotoxicity in melanoma and breast cancer and to present with negligible adverse effects in mice. This study investigates the anti-cancer activity of AJ-5 in alveolar and embryonal RMS. IC_50_ values of ≤ 0.2 µM were determined for AJ-5 and it displayed a favourable selectivity index of >2. Clonogenic and migration assays showed that AJ-5 inhibited the ability of RMS cells to survive and migrate, respectively. Western blotting revealed that AJ-5 induced levels of key DNA damage response proteins (γH2AX, p-ATM and p-Chk2) and the p38/MAPK stress pathway. This correlated with an upregulation of p21 and a G_1_ cell cycle arrest. Annexin V-FITC/propidium iodide staining revealed that AJ-5 induced apoptosis and necrosis. Apoptosis was confirmed by the detection of cleaved PARP and increased levels and activity of cleaved caspases-3, -7, -8 and -9. Furthermore, AJ-5 reduced autophagic flux as shown by reduced LC3II accumulation in the presence of bafilomycin A1 and a significant reduction in autophagosome flux *J*. Finally, pharmacokinetic studies in mice show that AJ-5 has a promising half-life and that its volume of distribution is high, its clearance low and its intraperitoneal absorption is good. Together these findings suggest that AJ-5 may be an effective chemotherapeutic with a desirable mechanism of action for treating drug-resistant and advanced sarcomas.

## Introduction

Rhabdomyosarcoma (RMS) originate in skeletal muscle and is the most common soft tissue sarcoma in children, adolescents and young adults^1,2^. It is classified as either embryonal (eRMS) or alveolar (aRMS), with each exhibiting distinct histological, molecular and pathological characteristics. Whereas eRMS affects predominantly infants and children, aRMS occur primarily in adolescents and young adults. Furthermore, eRMS comprises approximately 67% of RMS cases with common sites of tumorigenesis including the head, neck and genitourinary system. On the other hand, aRMS accounts for approximately 30% of RMS cases with arms and legs often being sites of primary disease^3–5^. RMS thus contributes to a considerable loss of years of life in comparison to other cancers as it largely affects children and adolescents.

The current standard treatment for patients with RMS is multimodal therapy consisting of local control with surgery and/or radiation therapy in conjunction with multi-agent chemotherapy^6^. Since the 1970s, disease control for patients with localized or completely resected RMS has been achieved with combinational chemotherapy most commonly including vincristine, dactinomycin (actinomycin D) and cyclophosphamide (VAC)^7^. However, this treatment regimen has had limited success for patients with regional spreading, incomplete resection or metastasis^8^. Furthermore, these drugs are associated with debilitating side effects including nausea, vomiting, fatigue, mouth ulcers, stomach ache, hair loss, bone marrow suppression, peripheral neuropathy and haemorrhagic cystitis^9–12^. Continued collaborative efforts to find more effective treatments with fewer side effects is therefore important, especially for metastatic RMS.

The use of transition metal complexes as potential therapeutics and in diagnostic medicine is of considerable importance. Platinum drugs, particularly cisplatin, are the mainstay of metal-based compounds in the treatment of cancer. However, due to dose-related adverse effects and multi-drug resistance associated with this line of therapy there has been an ongoing search for alternative metallic compounds with improved anti-cancer and pharmacokinetic properties and distinct mechanisms of action^13^. Palladium-based complexes have recently been reported to exert significant cytotoxicity in cancer cells^14^. Importantly, studies have shown that while both platinum and palladium compounds induce DNA damage, the palladium compounds exert a much greater degree of cytotoxicity in cancer cells^15,16^. Moreover, numerous palladium complexes exhibit strong cytotoxic effects in cells resistant to cisplatin^17^. Together these results suggest that palladium complexes may exert cytotoxicity through a mechanism different to that of platinum compounds and that they may prove to be less toxic and more effective for the treatment of aggressive cancers. Recently, AJ-5, a novel binuclear palladacycle complex with 1,2-bis(diphenylphosphino)ethane as co-ligand was shown to exert potent anti-tumour activity in advanced melanoma and breast cancer cells with very promising in vivo clearance of advanced melanoma^18,19^. In the present study we therefore explored these questions in RMS and other sarcoma subtypes. We show here for the first time that AJ-5 displays potent and selective cytotoxicity at sub-micromolar concentrations (≤0.2 µM) against aRMS and eRMS cells and inhibits their ability to survive, proliferate and migrate. We show that AJ-5 induces double-stranded DNA breaks (DSBs) which triggers the canonical DSBs ataxia telangiectasia mutated serine/threonine kinase (ATM)/checkpoint kinase 2 (Chk2) pathway as well as the p38/mitogen-activated protein kinase (MAPK) stress signalling pathway leading to a G_1_ cell cycle arrest, reduction in autophagic flux and induction of apoptosis. Furthermore, AJ-5 shows efficacy in a range of sarcoma subtypes and displays a promising pharmacokinetic profile in healthy mice. This study provides evidence that AJ-5 has promising therapeutic potential for the treatment of RMS and a range of other sarcoma subtypes.

## Results

### AJ-5 shows potent and selective cytotoxicity against RMS cells

**Fig. 1:**
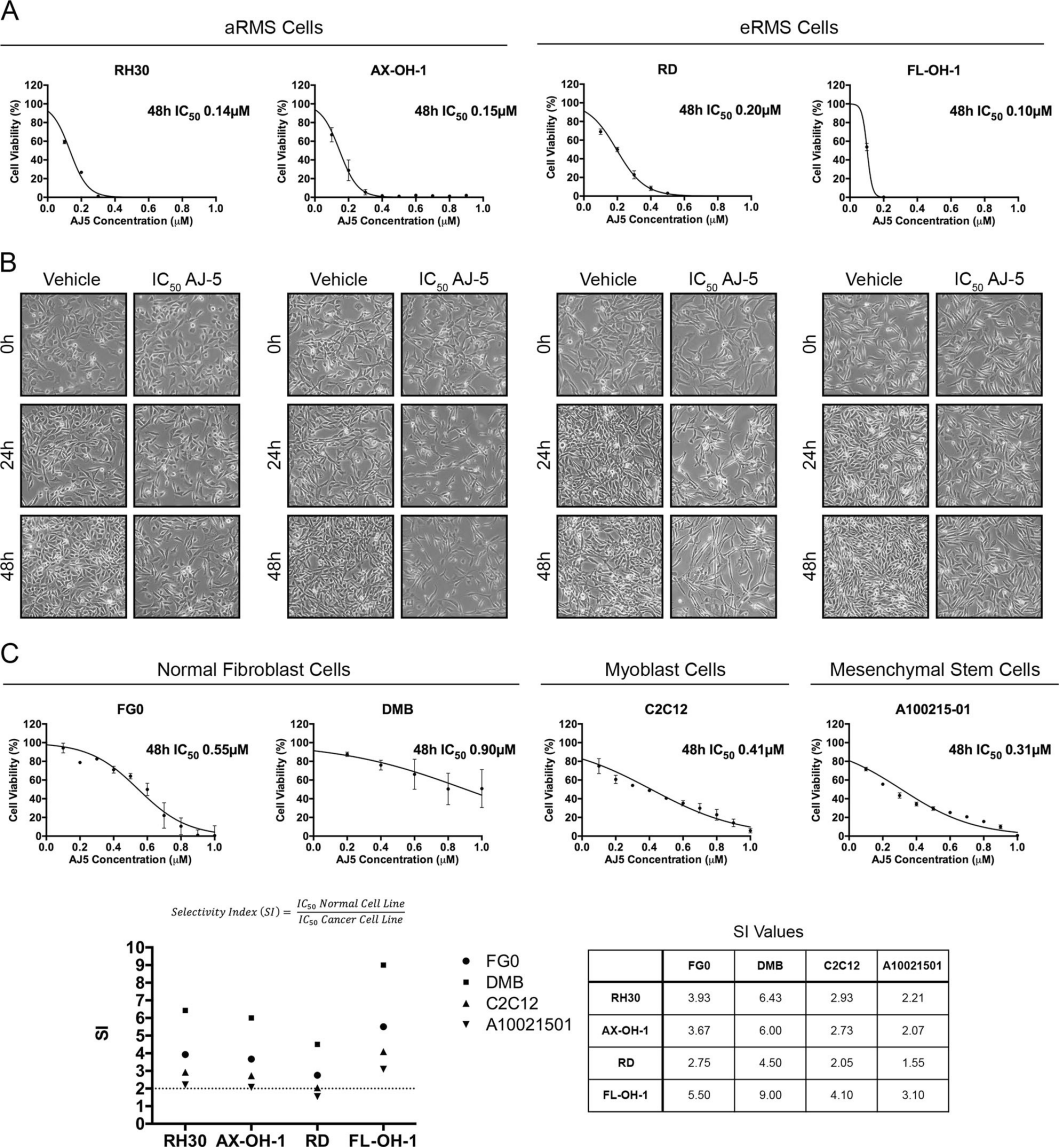
AJ-5 shows potent and selective cytotoxicity against aRMS and eRMS cells. **a** MTT cell viability assays of aRMS cell lines, RH30 and AX-OH-1, and eRMS cell lines, RD and FL-OH-1, treated with a range of AJ-5 concentrations (0.1–1.0 µM) or vehicle for 48 h. Graphs show mean cell viability as a percentage of vehicle control ± SEM for each concentration of AJ-5 determined from three independent experiments performed in quadruplicate. A curve was fitted to determine the IC_50_ concentration of AJ-5 for each cell line. **b** Representative light microscopy images (×200; EVOS XL AMEX1000 Core Imaging System) of aRMS and eRMS cell lines treated with their respective IC_50_ concentrations of AJ-5 or vehicle for 24 and 48 h. **c** MTT cell viability assays of non-malignant human fibroblast cell lines, FG0 and DMB, mouse myoblast cell line, C2C12, and mesenchymal stem cell line, A10021501, treated with AJ-5 and IC_50_ concentrations determined as described in **a** above. Selectivity indices (SIs) were determined for each RMS cell line by dividing the IC_50_ of each non-malignant cell line by the IC_50_ of each RMS cell line

Here we investigated the anti-tumour effects of the binuclear palladacycle complex, AJ-5, in RMS cells. To this end, MTT assays were performed on aRMS (RH30 and AX-OH-1 cell lines) and eRMS (RD and FL-OH-1 cell lines) cells treated with a range of AJ-5 concentrations (0.1–1.0 μM) for 48 h. IC_50_ concentrations of ≤0.2 µM were obtained for all cell lines (Fig. 1a). Light microscopy images of RMS cells treated for 24 and 48 h with their respective IC_50_ concentrations show the impact of AJ-5 on cell viability and morphology (Fig. 1b). To determine the selectivity of AJ-5 for RMS cells, IC_50_ values were determined in non-malignant fibroblasts (FG0 and DMB) and mouse myoblasts (C2C12). Results show that AJ-5 is less cytotoxic in the non-malignant cells with a selectivity index (SI) of >2 in all cases (Fig. 1c). Interestingly, there is growing evidence that mesenchymal stem cells are the cell of origin for specific sarcoma types including RMS^20,21^, and compared to the other non-malignant cells tested, the mesenchymal stem cells (A100021501) were the most sensitive to AJ-5.

### AJ-5 inhibits the ability of RMS cells to survive, proliferate and migrate

**Fig. 2:**
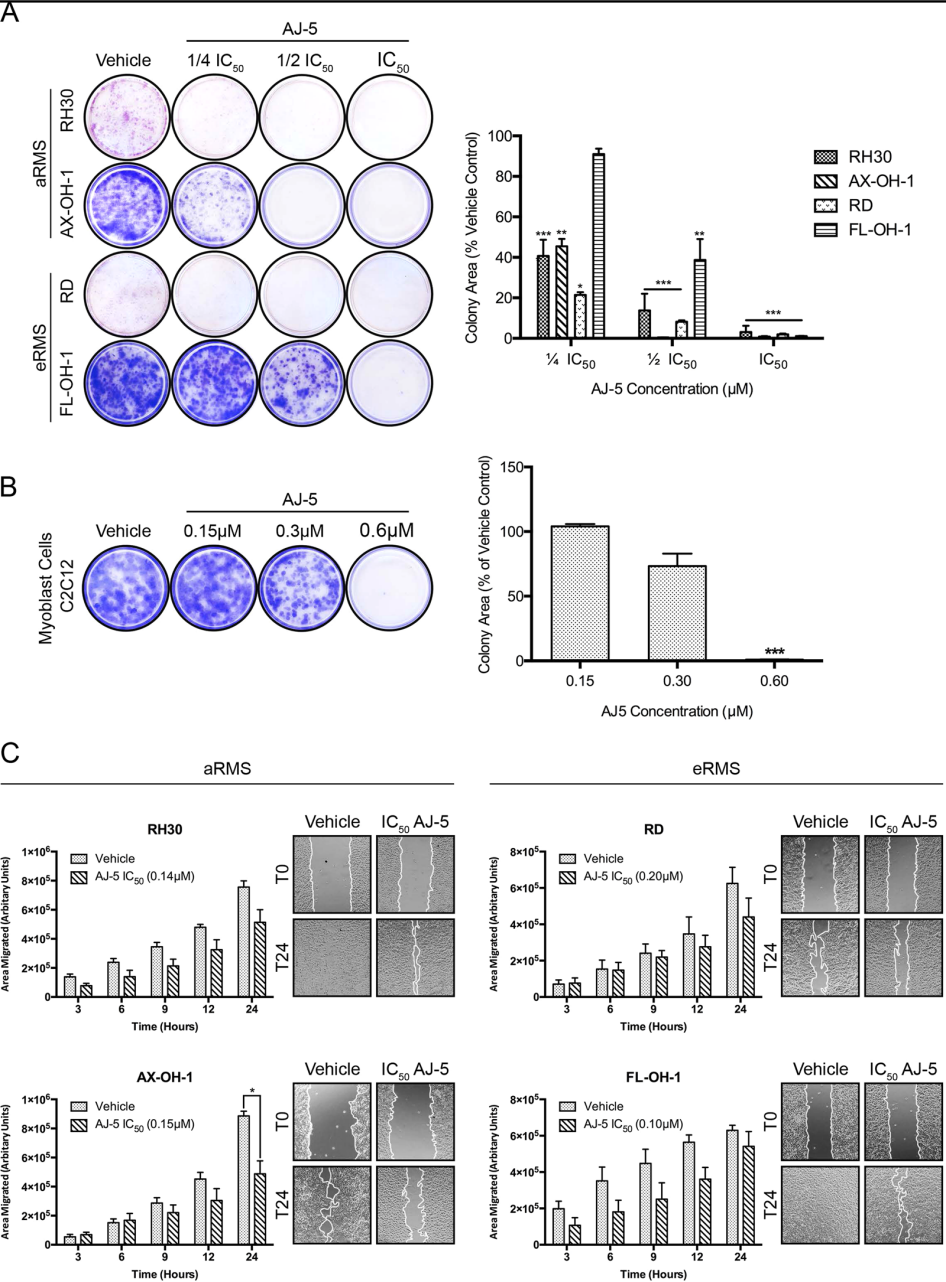
AJ-5 inhibits the ability of RMS cells to survive, proliferate and migrate. **a** Representative images and quantification of clonogenic assays of eRMS and aRMS cells treated with vehicle, ¼ IC_50_, ½ IC_50_ or IC_50_ concentrations of AJ-5 for 24 h and then replated at low densities in drug-free medium and left for 7–21 days for colonies to form. Colonies were stained with crystal violet and images from three independent repeats were quantified using the ImageJ plugin ColonyArea. The graph represents the mean colony area ± SEM of each treatment condition as a percentage of the vehicle control. **b** The same as **a** above for the non-malignant mouse myoblast cell line C2C12, but treated with vehicle, 0.15 µM, 0.3 µM, or 0.6 µM AJ-5. **c** Representative images (×200; EVOS XL AMEX1000 Core Imaging System) and quantification of scratch motility assays of eRMS and aRMS cells pre-treated with IC_50_ concentrations of AJ-5 or vehicle for 24 h and then replated at 100% confluency in drug-free medium. After cell adherence, a sterile 2 µL pipette tip was used to make a linear wound in the cell monolayer and cells were treated with 10 µg/mL mitomycin C to inhibit proliferation. Cells were imaged at 0, 3, 6, 9, 12 and 24 h post wound formation. Total area migrated was calculated by subtracting the wound area at each time point from the wound area at time 0 h, which is represented in the graphs as mean area migrated ± SEM pooled from three independent repeats. Data were analysed using GraphPad Prism 6.0 and a parametric unpaired *t*-test was performed where *p < 0.05, **p < 0.01, ***p < 0.001

To explore the potential impact of AJ-5 on the long-term fate of RMS cells, clonogenic assays were performed^22^. Figure 2a shows representative images and quantification of colonies formed over 7–21 days after 24 h of AJ-5 treatment. It is evident that AJ-5 significantly inhibits the ability of RMS cells to survive and proliferate. Indeed, for all except one RMS cell line tested, there was a significant decrease in colony area at ¼ IC_50_ treatment and only a few or no RMS cell colonies formed at IC_50_ concentrations. Importantly, the ability of the non-malignant C2C12 myoblasts to survive and proliferate was not significantly hindered after 24 h of 0.3 µM AJ-5 (Fig. 2b). This is at least 1.5 times higher than the IC_50_ values obtained for the RMS cells. To further explore the anti-cancer activity of AJ-5, RMS cells were exposed for 24 h to IC_50_ concentrations of the drug and scratch motility assays were performed. Fig. 2c shows that AJ-5 reduced the migratory ability of all RMS cells tested. Taken together, these data suggest that AJ-5 inhibits the ability of RMS cells to survive, proliferate and migrate.

To further explore the mechanisms by which AJ-5 exerts its cytotoxicity, the aRMS cell line RH30 and the eRMS cell line RD were used for further characterization.

### AJ-5 activates the DNA damage and p38 MAPK pathways in RMS cells

**Fig. 3:**
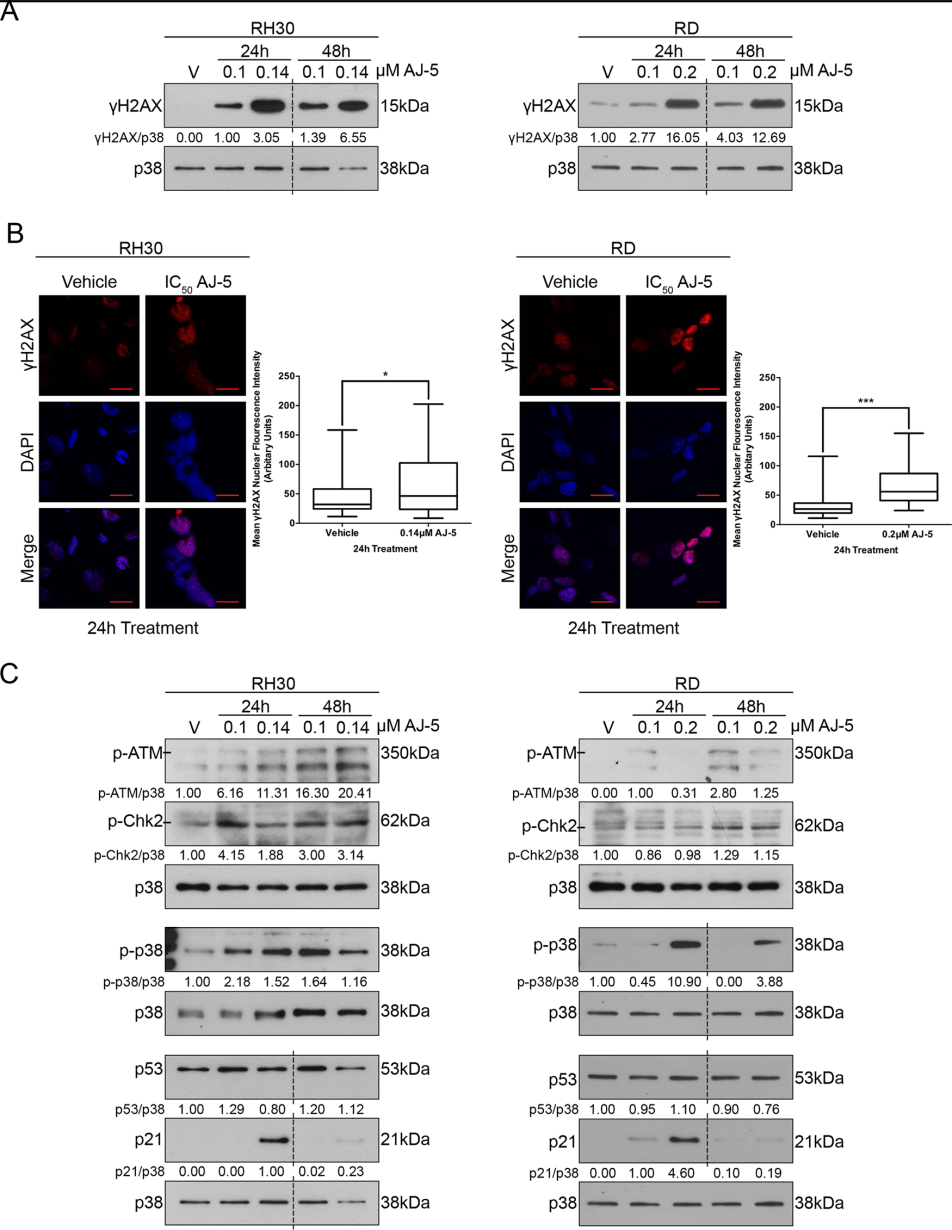
AJ-5 activates the DNA damage and the p38 MAPK pathways. **a** γH2AX protein levels detected by western blotting in RH30 (aRMS) and RD (eRMS) cells treated with vehicle (V), 0.1 µM or IC_50_ AJ-5 for 24 and 48 h. p38 was used as a loading control. Densitometry readings were obtained using ImageJ and protein expression levels are represented as a ratio of protein of interest/p38 normalized to the vehicle control sample (where possible). Blots are representative of at least two independent repeats. **b** Representative confocal immunofluorescence maximum intensity projection images (×630; Carl Zeiss LSM 510) of RH30 and RD cells treated with IC_50_ AJ-5 or vehicle for 24 h and γH2AX detected with a fluorophore-conjugated Cy3 secondary antibody. Nuclei of cells were stained with DAPI. Scale bar is 20 µM. Box plots represent quantification of γH2AX levels per treatment condition as mean nuclear Cy3 fluorescence from 20 fields of view from three independent repeats. Data were analysed using GraphPad Prism 6.0 and a parametric unpaired *t*-test was performed where *p < 0.05, **p < 0.01, ***p < 0.001. **c** Western blot analyses with antibodies to key DNA damage and stress signalling pathway proteins: p-ATM, p-Chk2, p-p38, p53, and p21. RH30 and RD cells were treated with AJ-5 and protein expression quantified as described above in **a**. Broken lines in western blots shown in this figure indicate where lanes not relevant were removed

γH2AX is a robust marker of DSBs^23^ and western blotting and immunocytochemistry revealed a dose-dependent increase in levels of γH2AX (Fig. 3a) and an accumulation of distinct γH2AX foci in the nuclei of AJ-5-treated cells (Fig. 3b), respectively. Furthermore, AJ-5 treatment led to increased levels of phosphorylated ATM and its downstream target CHK2 as well as phosphorylated p38 (Fig. 3c). AJ-5 thus activated the canonical DSB and p38 MAPK stress signalling pathways. Both of these pathways converge on p53, which ordinarily plays an important role in transcriptionally activating the cyclin-dependent kinase p21^24^. However, both the RMS cell lines used in this study harbour p53 mutations that result in high levels of inactive p53^25^. Importantly, p21 levels increased at the IC_50_ concentrations of AJ-5 and this response was particularly pronounced after 24 h of treatment (Fig. 3c). These results suggest that p21 levels increase in a p53-independent manner.

### AJ-5 triggers a G_1_ cell cycle arrest and induces apoptosis and necrosis

**Fig. 4:**
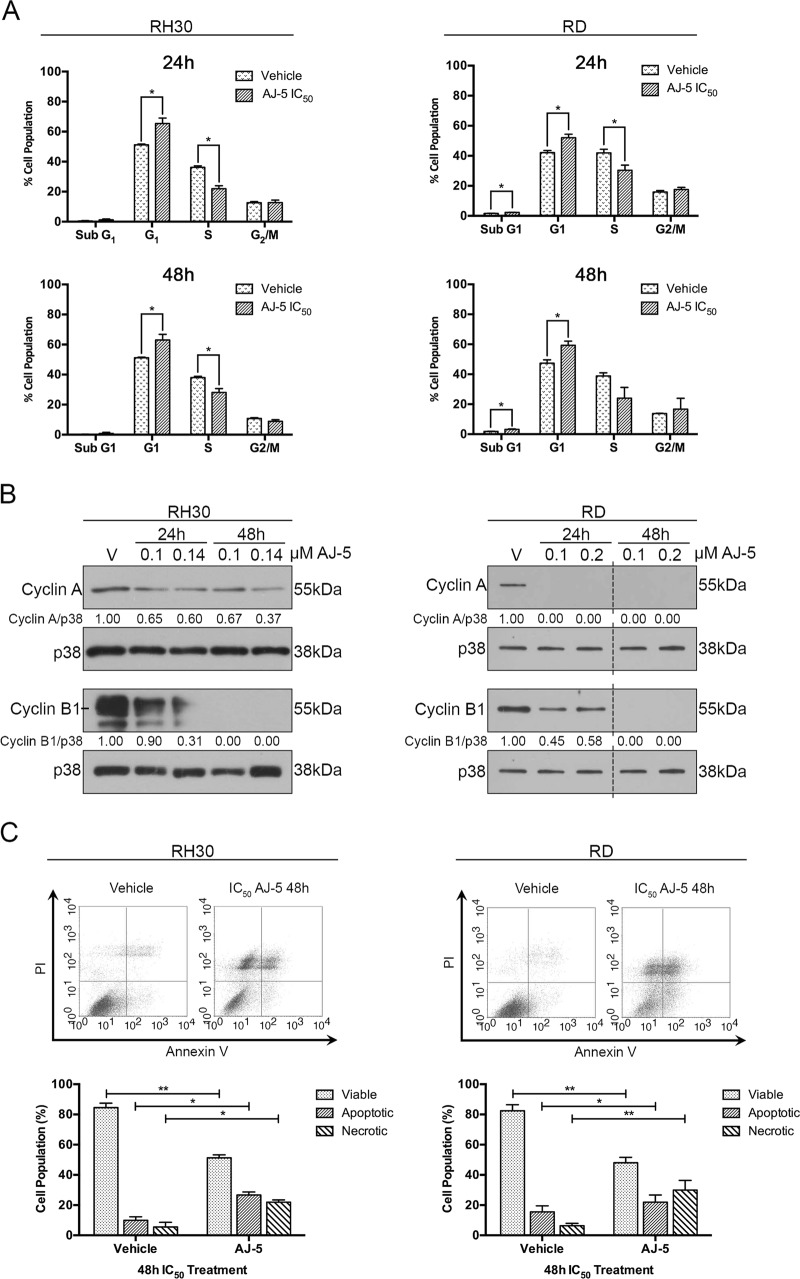
AJ-5 triggers a G1 cell cycle arrest and induces apoptotic and necrotic cell death in RH30 and RD cells. **a** Flow cytometry analyses of cells treated with vehicle or IC_50_ AJ-5 for 24 and 48 h. Graphs represent the mean proportion of cells ± SEM at each phase of the cell cycle pooled from three independent repeats. **b** Western blot analyses of protein harvested from cells treated as indicated and incubated with antibodies against cell cycle markers cyclin A and cyclin B1. p38 was used as a loading control. Densitometry readings were obtained using ImageJ and protein expression levels are represented as a ratio of protein of interest/p38 normalized to the vehicle control sample. Blots are representative of at least two independent repeats. Broken lines indicate where lanes not relevant were removed. **c** Flow cytometric analyses of cells treated with vehicle or IC_50_ AJ-5 and stained with Annexin V-FITC and PI. Graphs represent the mean ± SEM percentage of viable (lower left-hand quadrant), apoptotic (lower right-hand quadrant + upper right-hand quadrant) and necrotic (upper left-hand quadrant) cells from three independent experiments. For **a** and **c** data were analysed using GraphPad Prism 6.0 and a parametric unpaired *t*-test was performed where *p < 0.05, **p < 0.01, ***p < 0.001

To investigate the effect of AJ-5 on cell cycle progression, FACS analyses were performed on RMS cells treated with drug for 24 and 48 h. Results show that AJ-5 induces a G_1_ cell cycle arrest in both cell lines at the expense of the S phase (Fig. 4a). This correlated with a decrease in levels of cyclin A and cyclin B, which are required for progression through the S and G_2_/M phases, respectively^26^ (Fig. 4b). The sub-G_1_ peaks seen in Fig. 4a suggested that AJ-5 treatment leads to cell death. Indeed, Annexin V-FITC assays confirm that AJ-5 induces apoptotic and necrotic cell death (Fig. 4c).

### AJ-5 triggers the intrinsic and extrinsic apoptosis pathways in RMS cells

**Fig. 5:**
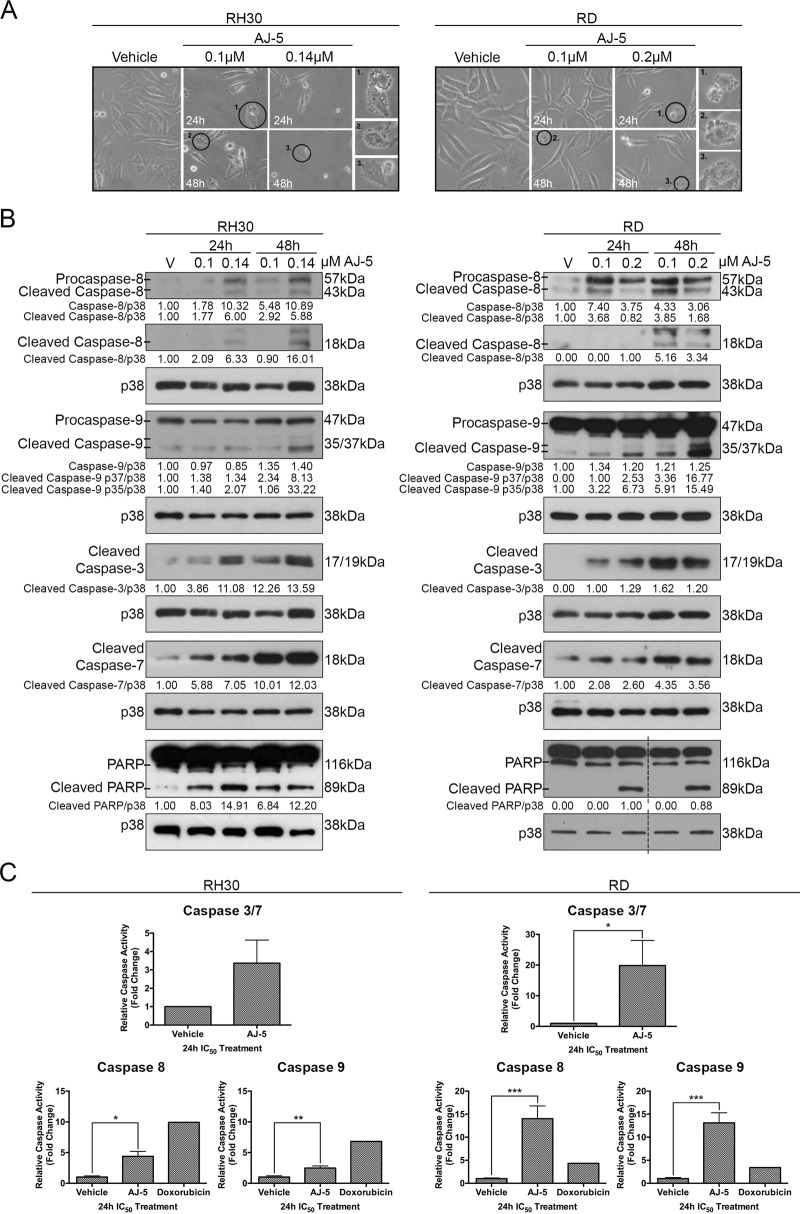
AJ-5 triggers the intrinsic and extrinsic apoptosis pathways in RMS cells. **a** Representative light microcopy images (×200; EVOS XL AMEX1000 Core Imaging System) showing the morphology of RH30 and RD cells treated with vehicle, 0.1 µM or IC_50_ AJ-5 for 24 and 48 h. Numbered circles correspond to magnified images on the right, which highlight characteristic apoptotic morphology including membrane blebbing and cell shrinkage. **b** Western blot analyses of protein harvested from cells treated as in **a** and incubated with antibodies as indicated. p38 was used as a loading control and densitometry readings were obtained using ImageJ. Protein expression levels are represented as a ratio of protein of interest/p38 normalized to vehicle control samples (where possible). Blots are representative of at least two independent repeats. Broken lines indicate where lanes not relevant were removed. **c** Caspase-Glo assays showing the enzymatic activity of caspase-3/7, caspase-8 and caspase-9 for cells treated with vehicle or IC_50_ AJ-5. Doxorubicin was included as a positive control for caspase-8 and -9 assays at the IC_50_ concentration (0.5 µM) established in the cell lines tested. Graphs represent the mean fold change of caspase activity ± SEM pooled from three independent repeats. Data were analysed using GraphPad Prism 6.0 and a parametric unpaired *t*-test was performed where *p < 0.05, **p < 0.01, ***p < 0.001

To confirm that AJ-5-induces apoptosis, RD and RH30 cells were treated with AJ-5 and biological, molecular and biochemical markers of apoptosis were investigated by light microscopy, western blotting and enzymatic Caspase-Glo assays, respectively. The results show that AJ-5 induced characteristic features of apoptosis including membrane blebbing and cell shrinkage (Fig. 5a). In addition, AJ-5 treatment led to increased levels and activity of cleaved caspase-8 and caspase-9, the effectors of the extrinsic and intrinsic pathways, respectively (Fig. 5b, c). This correlated with increased levels of the active (cleaved) forms of the executioner caspases-3/7 and their substrate poly (ADP-ribose) polymerase (PARP) (Fig. 5b, c)^27,28^. Interestingly, we observed an ~20 kDa band above the 18 kDa active cleaved caspase-8 fragment. A similar band has also been reported by other researchers and it has been suggested that it represents an intermediate product of procaspase-8 processing^29,30^. Importantly, caspases-8 and -9 were more robustly activated in RD cells treated with AJ-5 than those treated with IC_50_ doxorubicin, a drug commonly used in the treatment of RMS and used as a positive control in apoptosis assays (Fig. 5c). Together these results show that AJ-5 induces cell death in RMS cells, in part, through the extrinsic and intrinsic apoptotic pathways.

### AJ-5 induces several markers of autophagy in RMS cells

We next investigated whether AJ-5 induced autophagy because large vacuolar structures and acidic vesicles were observed in RMS cells treated with this drug (Supplementary Fig. S1A). To this end, western blotting and immunocytochemistry were performed with an antibody to microtubule-associated protein light chain 3 (LC3). Supplementary Figure S1B shows that LC3II (the autophagosomal membrane bound form of LC3) was induced in both RMS cell lines in a time- and dose-dependent manner. Furthermore, supplementary Fig. S1C shows distinct puncta, presumed to represent LC3II aggregates conjugated to autophagosomal membranes, in RD and RH30 cells from as early as 6 h after AJ-5 treatment.

### AJ-5 reduces autophagic flux in RD and RH30 cells

**Fig. 6:**
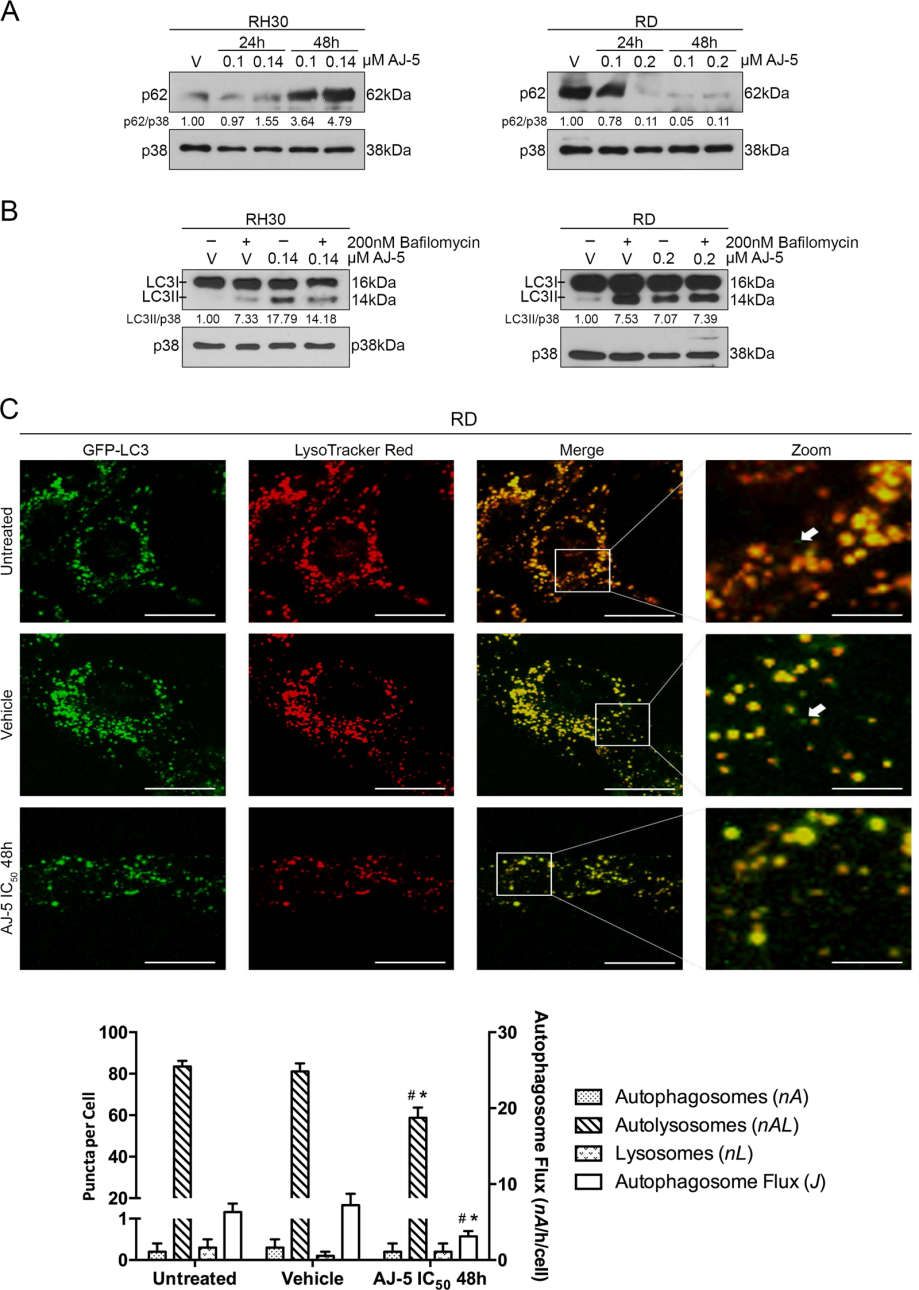
AJ-5 reduces autophagic flux in RD and RH30 cells. **a** Western blotting of p62/SQSTM1 protein levels in RH30 and RD cells treated with vehicle (V), 0.1 µM or IC_50_ AJ-5 for 24 and 48 h. **b** Western blotting showing LC3I and LC3II protein levels in RH30 and RD cells treated with vehicle (V) or IC_50_ AJ-5 for 24 h followed by 2 h of treatment with 200 nM bafilomycin A1. For western blots, p38 was used as a loading control and densitometry readings were obtained using ImageJ. Protein expression levels are represented as a ratio of protein of interest/p38 normalized to vehicle control sample. Blots are representative of at least two independent repeats. **c** Representative single-cell fluorescence maximum intensity projection micrographs (×630; Carl Zeiss LSM 780; scale bar is 20 µM) and pool size quantification of autophagy pathway intermediates: autophagosomes (GFP-LC3, *nA*) (indicated with white arrows in the merged image), autolysosomes (LysoTracker Red, *nAL*) and lysosomes (merged, *nL*). Autophagosome flux *J* was calcuclated. Data were analysed using GraphPad Prism 6.0 and a parametric unpaired *t*-test was performed *p < 0.05, **p < 0.01, ***p < 0.001. ^#^ compared to untreated control, * compared to vehicle control

The detection of LC3II alone does not distinguish between autophagy induction and cessation in the downstream steps of autophagy. We therefore next determined the impact of AJ-5 treatment on autophagic flux by measuring the levels of p62, a receptor of polyubiquitinated proteins. The rationale for this is based on observations that upon autophagy induction, p62 is incorporated within an autophagosome and degraded within an autolysosome^31^. The turnover of p62 is therefore considered an indicator of autophagic flux. Interestingly, Fig. 6a shows that whereas AJ-5 appears to reduce autophagic flux in RH30 cells, it increases autophagic flux in RD cells. To confirm the above data, levels of LC3II were determined by western blotting in the presence and absence of bafilomycin A1 which blocks the autophagy pathway by inhibiting fusion of autophagosomes with lysosomes^32^. An accumulation of LC3II in the presence of this inhibitor is therefore considered evidence of autophagic flux. Consistent with our p62 data in RH30 cells, AJ-5 treatment followed by bafilomycin A1 exposure led to decreased levels of LC3II (Fig. 6b). Interestingly, contrary to our p62 data in RD cells, in the presence of bafilomycin A1, AJ-5 treatment led to a negligible change in LC3II compared with vehicletreated cells (Fig. 6b). We therefore further investigated the impact of AJ-5 on autophagic flux in the RD cell line by performing single-cell autophagosome flux analyses. Our results reveal that RD cells are characterized by a predominant autolysosomal pool (*nA* = 83 autolysosomes/cell), with only a very minor pool of autophagosomes and lysosomes per cell (Fig. 6c). Moreover, the cells are characterized by a basal autophagosome flux *J* of 6.3 autophagosomes per hour per cell. Upon AJ-5 treatment, however, both autolysosome pool size as well as autophagosome flux significantly decreased. This suggests that AJ-5 negatively impacts the rate of autophagosome synthesis, which supports the data showing that in the presence of bafilomycin A1, AJ-5 treatment does not lead to LC3II accumulation (Fig. 6b). Together these data suggest that AJ-5 reduces autophagic flux in RH30 and RD cells.

### AJ-5 is cytotoxic in a range of sarcoma subtypes

To investigate if the therapeutic potential of AJ-5 could be extended to other sarcoma subtypes, chondrosarcoma (SW1353), liposarcoma (SW872), synovial sarcoma (SW982), fibrosarcoma (HT1080) and osteosarcoma (MG-63) cells were treated with the drug as described earlier and MTT assays were performed. Our results show that an IC_50_ of <0.3 µM was obtained for all the sarcoma cell lines tested (Supplementary Fig. S2A) and a favourable SI of >2 was achieved when calculated relative to the combined IC_50_ values for the normal fibroblasts (FG0 and DMB). However, a sub-optimal SI between 1 and 1.5 was obtained when the IC_50_ values for the sarcoma cells were expressed relative to the mesenchymal stem cells (A10021501) (Supplementary Fig. S2B). This raises the interesting possibility that AJ-5 may be effective against the cells of origin of these sarcoma subtypes which may be of therapeutic benefit. Furthermore, clonogenic assays reveal that as little as a ¼ IC_50_ concentration of AJ-5 significantly reduced the ability of cells of all sarcoma subtypes to survive and proliferate (supplementary Fig. S2C). AJ-5 therefore shows potent selective cytotoxicity against a number of diverse sarcoma subtypes and may therefore have broad therapeutic potential.

### Pharmacokinetic (PK) profile of AJ-5 in healthy mice

Given its importance to the drug discovery process, we next tested the in vivo PK profile of AJ-5 in whole blood of MF1 mice following a single dose of 2 mg/kg intravenous (IV), 2 mg/kg intraperitoneal (IP) or 20 mg/kg oral (PO) for a period of 24 h. The blood concentration–time curve of AJ-5 over a 24 h period and the calculated PK parameters are shown in Supplementary Fig. S3 and Table S1. For IV administration, AJ-5 illustrated a long half-life (>10 h), which is most likely due to the low clearance (9.2 mL/min/kg) and a high volume of distribution (8.8 L/kg). The exposure of AJ-5 following the IP dose of 2 mg/kg was eight-fold higher compared to the PO dose of 20 mg/kg with an area under the curve of 88 and 11 min.µM/L, respectively. The data obtained for the IP group in healthy mice correlated well with our previously observed in vivo efficacy of AJ-5 in advanced melanoma^18^.

## Discussion

**Fig. 7:**
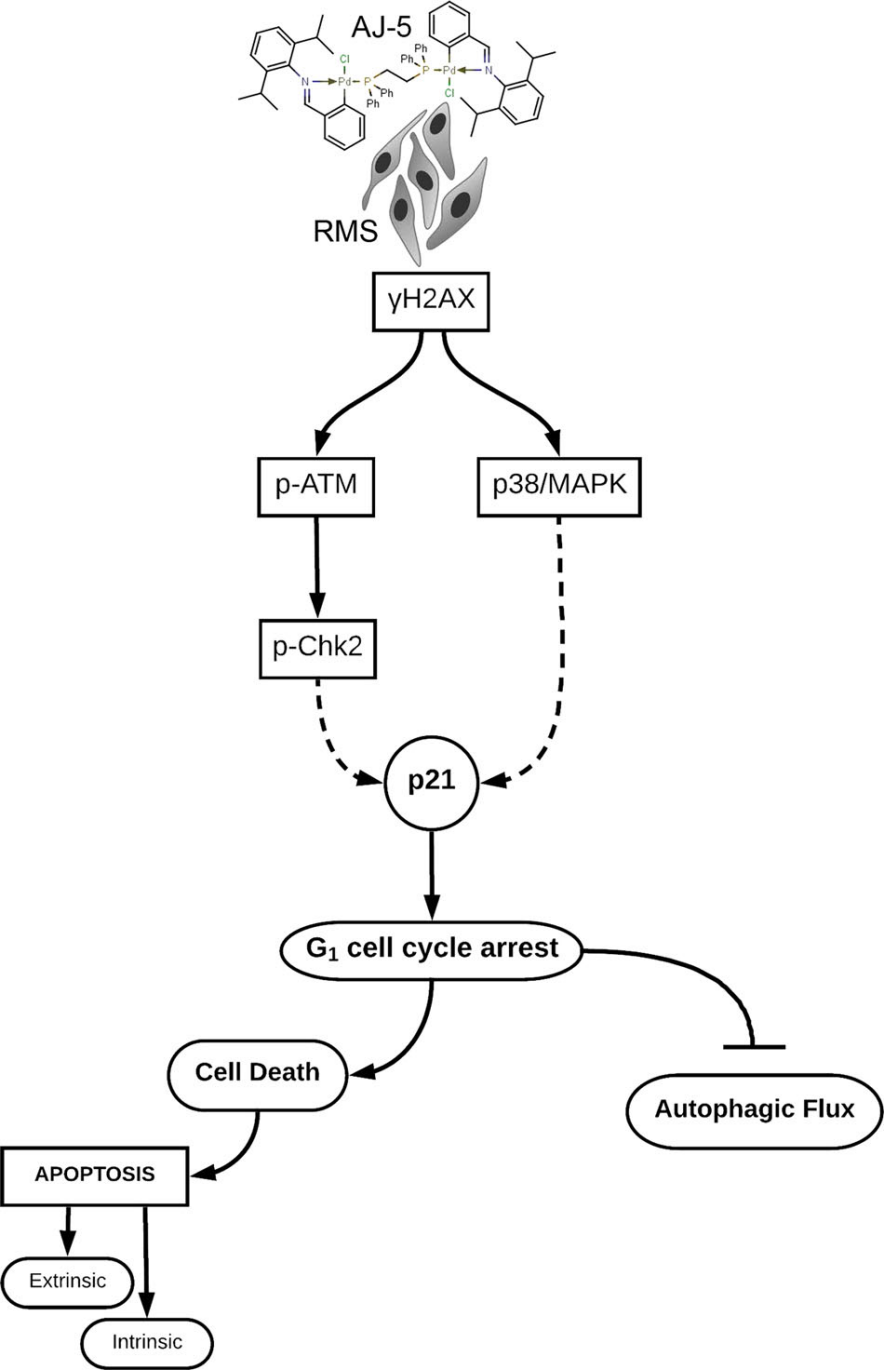
Proposed model for AJ-5 in RMS. At low concentrations (≤0.2 µM) in aRMS and eRMS cells AJ-5 induces DSBs leading to increased levels of γH2AX and activation of both the canonical DSB pathway (ATM/Chk2) and the p38/MAPK stress signalling pathway. This results in a p21-induced G_1_ cell cycle arrest followed by the activation of the extrinsic and intrinsic apoptotic pathways and a reduction in autophagic flux

RMS is the most common soft tissue sarcoma found in children and adolescents and while the current treatment for localized tumours results in a high overall survival rate, the chemotherapeutic agents used are associated with debilitating adverse effects^10,33–36^. Moreover, more than 15% of patients present with metastatic disease and there has been limited improvement in the treatment of patients with recurrent and metastatic disease^37–41^. The current study provides several lines of evidence that the binuclear palladacycle AJ-5 may be a promising chemotherapeutic to treat aRMS and eRMS cells as well as a range of other sarcoma subtypes. Furthermore, we provide novel and interesting mechanisms by which AJ-5 functions (see Fig. 7).

Platinum compounds such as cisplatin have been used successfully as anti-tumour agents but they have been associated with severe toxicity and tumour drug resistance^42^. Palladium compounds have been proposed as more effective alternatives because they appear to be more active at lower concentrations with less severe side effects^43,44^. Cisplatin is occasionally used to treat RMS^6^ and was reported to have an IC_50_ of >0.5 µM after 4–5 days of treatment in a range of RMS cell lines including RD and RH30 cells^45,46^. Our study shows that after only 2 days of AJ-5 treatment, IC_50_ values of ≤0.2 µM were obtained in the RD and RH30 cells. This suggests that AJ-5 displays more potent anti-cancer activity at much lower concentrations over a shorter period than cisplatin in these RMS cells. Furthermore, we show that AJ-5 displays a selectivity index of ≥2, which suggests that it has specificity for RMS cells^47,48^. Indeed, whereas AJ-5 treated RMS cells were unable to form any colonies, mouse myoblasts treated with 1.5–3 times higher concentrations formed colonies with areas comparable to that obtained for vehicletreated cells (Fig. 2a). Furthermore, mesenchymal stem cells from different sources are thought to represent the target cell of origin for a variety of human sarcomas and our cell viability assays (Fig. 1c) show that the A100021501 mesenchymal stem cells were the most sensitive non-malignant cell line to AJ-5 treatment^20^. This suggests that AJ-5 may have the ability to selectively target the sarcoma-initiating cell which could prove to be advantageous and more efficacious than current therapies. Taken together these findings support evidence that, compared to platinum compounds, palladium complexes display superior anti-cancer activity and that they may associate with fewer side effects.

While tumours initially respond to proapoptotic therapies, they frequently acquire the ability to bypass the apoptotic pathway and develop drug resistance leading to tumour recurrence^49,50^. It is thus predicted that drugs that can trigger alternative or multiple programmed cell death (PCD) pathways such as autophagy (PCD type II) and programmed necrosis (PCD type III) would be more efficacious^51^. The annexin V-FITC assays in Fig. 4c reveal that AJ-5 induces not only a significant apoptotic population but also a significant cell population which could represent necrosis, programmed necrosis (necroptosis) and/or secondary necrosis which occurs when there is insufficient clearance of apoptotic cells^49,52^. Interestingly, we have preliminary western blot data that show that AJ-5 treatment causes an increase in phosphorylated receptor-interacting serine/threonine protein kinase 3 (RIP3), a critical component of necroptosis, as well as increased levels of active (phosphorylated) mixed lineage kinase domain-like protein (MLKL), a key executioner of necroptosis (data not shown). The possibility that AJ-5 may induce necroptosis is exciting; however, to confirm this annexin V-FITC assays of AJ-5-treated cells in the presence of necrostatin-1 and the pan-caspase inhibitor Z-VAD-fmk will need to be performed.

Here we show for the first time that AJ-5 reduces autophagic flux in both aRMS and eRMS cells. This is an important finding because autophagy has been reported to promote cell survival and chemotherapeutic drug resistance in a number of sarcomas, including RMS, and thus autophagy has been identified as a drug target to treat sarcomas^50,51,53^. For example, the autophagy inhibitor, chloroquine, enhanced RMS cell death induced by ciclopirox olamine, bortezomib and 17-DMAG and stimulation of autophagy by rapamycin prevented this^54,55^. Consistent with our findings, palladium nanoparticles have recently been shown to block autophagic flux in Hela cells as shown by increased LC3II/I ratios, reduced degradation of p62 and autophagosome accumulation^56^. It is important to note that AJ-5 induces autophagy PCD in melanoma and breast cancer cells, which suggests that the effect of palladium complexes on autophagy may be cancer type specific.

In conclusion, we show that AJ-5 may be a promising chemotherapeutic to treat aRMS and eRMS because it displays potent and selective cytotoxicity at sub-micromolar concentrations and inhibits the ability of RMS cells to survive, proliferate and migrate. Importantly, AJ-5 induces apoptosis and reduces autophagic flux and it displays a favourable pharmacokinetic and safety profile.

## Materials and methods

### Cell culture

RH30 human aRMS cells (kindly provided by Associate Professor Judith Davie, Southern Illinois University), AX-OH-1 human aRMS and FL-OH-1 human eRMS cells (kindly provided by Professor Stefan Bath, University of Cape Town) were cultured in Roswell Park Memorial Institute Medium (RPMI)-1640 (Sigma Aldrich, Missouri, USA). RD human eRMS cells (ATCC^**®**^ CCL-136**™**), FG0 and DMB human skin fibroblasts (kindly provided by Associate Professor Denver Hendricks, University of Cape Town), mouse myoblast cells (ATCC^**®**^ CRL-1772**™**), human mesenchymal stem cells A10021501 (kindly provided by Professor Michael Pepper, University of Pretoria and confirmed to meet the criteria to be defined as mesenchymal stem cells as set out by the Mesenchymal and Tissue Stem Cell Committee of the International Society for Cellular Therapy^57^), HT1080 human fibrosarcoma cells (ATCC^**®**^ CCL-120**™**), SW1353 human chondrosarcoma cells (ATCC^**®**^ HTB-94**™**), SW982 human synovial sarcoma cells (ATCC^**®**^ HTB-93**™**), SW872 human liposarcoma (ATCC^**®**^ HTB-92**™**) and MG-63 human osteosarcoma cells (kindly provided by Associate Professor Philippa Hulley, University of Oxford) were cultured in Dulbecco’s modified Eagle’s medium (Sigma Aldrich). All culture medium was supplemented with 10% heat-inactivated foetal bovine serum, 100 U/mL penicillin and 100 μg/mL streptomycin. Cells were maintained at 37 °C in a 95% air and 5% CO_2_ humidified incubator. Medium was replaced every 2 to 3 days and cells were routinely subjected to mycoplasma tests. Only mycoplasma free cells were used in experiments. Cell morphology was monitored using an Olympus CKX41 inverted microscope (MSAC Ltd, UK) and imaged with an EVOS™ XL AMEX1000 Core Imaging System (Thermo Fisher Scientific, Massachusetts, USA).

### Cell treatments

AJ-5, synthesized as previously described^18^, was dissolved in dimethyl sulfoxide (DMSO) (D8418; Sigma Aldrich) to give a 5 mM AJ-5 stock which was stored at room temperature (RT) and used within 5 days. AJ-5 was diluted in cell culture medium to achieve the desired final concentration and a vehicle control (DMSO) of the same concentration was prepared simultaneously. Cells were treated at a confluency of 60%. For western blot experiments to determine autophagy flux, cells were treated with 200 nM bafilomycin A1 (B1793; Sigma Aldrich) for 2 h post AJ-5 treatment. Doxorubicin (z/26/0167; Teva Pharmaceutical Industries Ltd, Israel) was used as a positive control in Caspase-Glo experiments at a concentration of 0.5 µM.

### Cell viability assays

Cells were seeded in 96-well plates and treated the next day with a range of AJ-5 concentrations (0.1–1.0 μM) or vehicle (1.0 μM DMSO) for 48 h. Cell viability was measured using the 3-(4,5-dimethylthiazol-2-yl)-2,5-diphenyl-trazolium bromide (MTT) assay (11465007001; Sigma Aldrich) according to the manufacturer’s instructions. Mean cell viability was calculated as a percentage of the mean vehicle control. At least three independent experiments in quadruplicate were performed from which the half maximal inhibitory concentration (IC_50_) was determined using GraphPad Prism version 6.0 (GraphPad Software, California, USA). The selectivity index (SI) was determined by dividing the IC_50_ of a normal cell line by the IC_50_ of a cancer cell line.

### Clonogenic assays

Cells were seeded in 6-well plates and treated the following day with IC_50_, ½ IC_50_, ¼ IC_50_ AJ-5 and vehicle. After 24 h, 800–4000 cells were seeded in 35 mm dishes in drug-free medium. Formation of colonies were monitored and after 7–21 days cells were fixed with 3:1 methanol:acetic acid and stained with 0.5% crystal violet (Sigma Aldrich) in 100% methanol. Colonies were imaged and percentage colony area was determined using ImageJ v1.50i^58^ and the plugin ColonyArea^59^. Colony area was determined for each drug concentration and expressed as a percentage of vehicletreated control.

### Scratch motility assay

Cells were seeded in 6-well plates and treated with IC_50_ AJ-5 and vehicle for 24 h. Cells were then collected, counted and replated to achieve 100% confluency in a 24-well plate. The following day a sterile 2 µL pipette tip was used to make a vertical scratch in the cell monolayer of each well and the cells treated with 10 µM mitomycin C (M4287; Sigma Aldrich) to inhibit proliferation. Cells were imaged at 0, 3, 6, 9, 12 and 24 h post wound formation and ImageJ v1.50i^58^ was used to calculate the area of the scratch. The total areas migrated was determined by subtracting the area for a specific time point from the area measured at 0h.

### Western blot analyses

Cells were harvested after exposure to IC_50_ AJ-5, 0.1 µM AJ-5 or vehicle (DMSO) for 24 and 48 h. Cells were lysed at 4 °C in whole-cell lysis buffer (0.125 M Tris-HCl pH 6.8, 4% SDS, 0.2% glycerol, 0.1% β-mercaptoethanol and a pinch of bromophenol blue) and boiled for 10 min. Proteins were resolved by SDS-PAGE (6–15% gels) and transferred to Hybond ECL membranes (Amersham, UK). The membranes were incubated with primary antibodies: rabbit polyclonal antibodies to phospho-histone H2A.X (Ser139) (#2577), phospho-chk2 (Thr68) (#221), phospho-p38 MAPK (Thr180/Tyr182) (#9211), cleaved caspase-3 (Asp175) (#9661), PARP (#9542), caspase-9 (#9502), LC3B (#2775), p38 MAPK (#9212), rabbit monoclonal antibody to cleaved caspase-7 (Asp198) (D6H1) (#8438), mouse monoclonal antibodies to phospho-ATM (Ser1981) (D6H9) (#5883), Cyclin B1 (V152) (#4135), Caspase-8 (1C12) (#9746), SQSTM1/p62 (D5L7G) (#88588) from Cell Signaling Technology (Massachusetts, USA); mouse monoclonal antibody to p53 (DO-1) (sc-126), rabbit polyclonal antibodies to p21 (C-19) (sc-397), cyclin A (H-432) (sc-751), cyclin B1 (H-433)(sc-752), PARP-1 (H-250) (sc-7150) from Santa Cruz Biotechnology (Texas, USA); rabbit polyclonal antibody to p38 MAP kinase (M0800) from Sigma Aldrich. After primary antibody incubation, membranes were incubated with goat anti-rabbit or goat anti-mouse HRP-conjugated secondary antibodies (Bio-Rad Laboratories, California, USA). Antibody reactive proteins were visualized by enhanced chemiluminescence using SuperSignal West Pico Chemiluminescent Substrate Kit (Thermo Fisher Scientific) or WesternBright ECL HRP Substrate Kit (Advansta, California, USA). Densitometry readings were obtained using ImageJ v1.50i^58^ and protein expression levels were represented as a ratio of protein of interest/p38 loading control normalized to the vehicletreated control sample where appropriate. All blots are representative of at least two independent repeats.

### Immunofluorescence

Cells were plated on glass coverslips and treated with IC_50_ AJ-5 or vehicle. Cells were fixed with ice-cold 100% methanol at −20 °C for 5 min followed by blocking and permeabilization with 0.2% Triton-X-100/5% bovine serum albumin in phosphate-buffered saline (PBS) for 30 min at RT. Slides were incubated with antibodies against phospho-histone H2A.X (#2577) (1:500) or LC3B (#2775) (1:200) (Cell Signaling Technology) in blocking buffer overnight at 4 °C. After PBS washes coverslips were incubated with donkey anti-rabbit or donkey anti-mouse Cy3-conjugated secondary antibodies (Jackson ImmunoResearch Laboratories Inc., Pennsylvania, USA) diluted 1:1000 for 1.5 h in blocking buffer at RT. A secondary-antibody-only control was always included in these experiments. Cells were then counterstained with Hoechst (33342; Invitrogen, California, USA) for 10 min at RT in the dark, washed with PBS and then coverslips were mounted onto glass slides using Mowiol mounting medium. Cells were imaged with an LSM 510 confocal microscope (Carl Zeiss, Germany) using a Plan-Apochromat ×63/1.40 oil DIC objective. Multiple z layers were acquired with 1 µM step width. Images were processed using ZEN 2012 imaging software (Carl Zeiss) and maximum intensity projections were generated. For quantification, mean fluorescence was measured from at least 20 fields of view per treatment condition and pooled from three independent repeats.

### Cell cycle analysis

Cells were seeded and treated the following day with IC_50_ AJ-5 and vehicle for 24 and 48 h, trypsinized, washed with PBS, counted and permeabilized in 70% EtOH at −20 °C overnight. Cells were pelleted and treated with 50 μg/mL RNase (Fermentas, Massachusetts, USA) in PBS at 37 °C for 15 min and then stained with propidium iodide (PI). A minimum of 50 ,000 cells/sample were subjected to analysis using a Becton Dickinson FACSCalibur flow cytometer (Becton Dickinson, New Jersey, USA) with a 488 nm coherent laser. The data were acquired using CellQuest Pro version 5.2.1. software (Becton Dickinson) and the analyses were done using ModFit version 2.0. software (Verity Software House Inc, Maine, USA).

### Annexin V-FITC assays

Cells were seeded, trypsinized, washed and counted as described for cell cycle analysis. The FITC Annexin V/Dead Cell Apoptosis kit (V13242; Thermo Fisher Scientific) was used according to the manufacturer's instructions. A negative control of unstained cells and positive controls of cells treated with 3% formaldehyde for 30 min on ice and stained with either PI or Annexin V were used to gate the flow cytometer for viable, early apoptotic, late apoptotic, and necrotic cell populations. Samples were subjected to FACS analyses as described for cell cycle analysis. Data were obtained and analysed using CellQuest Pro version 5.2.1. software.

### Caspase activity assays

Cells were seeded and treated for 24 h with IC_50_ AJ-5, vehicle or 0.5 µM (IC_50_) doxorubicin which was included and as a positive control. Cells were trypsinized, washed and counted and caspase-8, -9, and -3/7 activity was measured using the appropriate Caspase-Glo® assay kits (Promega, Wisconsin, USA) according to the manufacturer’s instructions. Luminescence was measured 45 min after incubation with Caspase Glo® reagent using a Luminoscan Ascent luminometer (Thermo LabSystems Inc., Massachusetts, USA). Luminescence signal for treated samples were normalized to vehicle-treated samples to express a fold change increase in caspase activity.

### Supravital staining with acridine orange

Cells were seeded on glass coverslips and treated for 24 h with IC_50_ AJ-5 or vehicle and then stained with 2 µg/mL acridine orange (Michrome No. 714; Edward Gurr Ltd, UK), which was added directly to the medium, for 15 min at RT in the dark. Coverslips were mounted on glass slides and visualized and imaged as described for immunofluorescence.

### Single-cell autophagosome flux analyses

The autophagy steady-state variables were measured in RD cells treated with IC_50_ AJ-5 for 48 h. The autophagosome flux was calculated from the rate of autophagosome accumulation as previously described^60^. Briefly, this was determined after the complete inhibition of fusion between autophagosomes and lysosomes using 400 nM bafilomycin A1 (B0025; LKT Laboratories Inc., Minnesota, USA).

Cells were transfected with a GFP-LC3 construct using Lipofectamine 3000 (L3000001; Thermo Fisher Scientific) according to the manufacturer’s instructions and maintained for 2 days in order to achieve high levels of GFP-LC3 expression. Thereafter, cells were harvested by trypsinization and seeded into an eight-chamber coverslip-based dish (155411 Nunc™ Lab-Tek™; Thermo Fisher Scientific) to achieve 60% confluency after which they were treated with IC_50_ AJ-5 for 48 h.

Autophagosome, autolysosome and lysosome pool size was assessed by supplementing the culture media with 25 nM LysoTracker Red (L7528; Thermo Fisher Scientific) 2 h prior to image acquisition. An LSM 780 confocal with ELYRA S.1 Superresolution platform (Carl Zeiss) was used for image acquisition. The eight-chamber dish was placed in a heated chamber maintained at 5% CO_2_ that encased the microscope objective. An LCI Plan-Apochromat ×63/1.4 oil DIC M27 objective was used and samples were excited with an Argon multiline laser 25 mW at 488 and 514 nm with appropriate beam splitters and GaAsP detector 32+2 PMT. Multiple z layers were acquired with 1 µM step width. Laser power and electron gain were set to achieve an optimal signal to noise ratio without causing photo-toxicity. Images were processed using ZEN 2011 imaging software (Carl Zeiss) and the maximum intensity projections were exported to Fiji software^61^ for further analysis^60^.

### Pharmacokinetics studies of AJ-5 in healthy mice

All studies and procedures were conducted with prior approval of the Animal Ethics Committee of the University of Cape Town (Protocol 012/049) in accordance with the South African National Standard (SANS 10386:008) for the Care and Use of Animals for Scientific Purposes^62^, and guidelines from the Department of Health^63^.

Pharmacokinetic parameters of AJ-5 were determined after IV, IP and PO dosing to MF1 nude mice randomly divided into groups. AJ-5 was prepared in the respective formulations immediately prior to dosing. For the IV group, the compound was made up in a mixture of 10% DMSO and 90% of an IV mixture, which consisted of 60% propylene glycol, 10% ethanol and 30% polyethylene glycol. For the PO group, AJ-5 was dissolved in DMSO and a hydroxypropylmethylcellulose solution (0.5% in water) containing 0.2% Tween 80 in a ratio of 1:9. For the IP group, AJ-5 was dissolved in 10% DMSO and 90% phosphate buffer solution. Mice were permitted access to food and water ad libitum. AJ-5 was administered (100 μL) by oral gavage at a dose of 20 mg/kg (*n* = 3), intravenously into the penile dorsal vein at a dose of 2 mg/kg (*n* = 2) and intraperitoneally at a dose of 2 mg/kg (*n* = 3).

Blood samples (20 μL per sample) were collected by tail tip bleeding in lithium heparin tubes at predetermined time points to evaluate the kinetic profile over 24 h. Samples were stored at −80 °C until analysis. Blood samples together with calibration standards and quality controls were quantified using inductively coupled plasma mass spectrometry. All results reported are for palladium (Pd). Pharmacokinetic parameters of the AJ-5 complex were determined using non-compartmental analysis in PK Solutions 2.0 (Summit Research Systems, Montrose CO, USA).

### Statistical analyses

All data were obtained from at least three independent experiments (unless otherwise stated) with error bars representing standard error of the mean (SEM). Data were analysed using GraphPad Prism version 6.0 (GraphPad Software) and a parametric unpaired *t*-test was performed. Significance was accepted at *p < 0.05,**p < 0.01 and ***p < 0.001.

## Supplementary information

Pharmacokinetic parameters of AJ-5 obtained from whole blood of healthy MF1 mice

AJ-5 induces several markers of autophagy in RMS cells

AJ-5 is cytotoxic in a range of sarcoma subtypes

Whole blood concentration of AJ-5 over 24h

Supplemental Material File #1
