# Supplementary material for: The palladacycle complex AJ-5 induces apoptotic cell death while reducing autophagic flux in rhabdomyosarcoma cells
Source: Cell Death Discov. 2019 Jan 28;5:60. doi: 10.1038/s41420-019-0139-9 (PMC6349869; doi:10.1038/s41420-019-0139-9)
Supplement: Supplementary file 1 — Pharmacokinetic parameters of AJ-5 obtained from whole blood of healthy MF1 mice [file 41420_2019_139_MOESM1_ESM.docx]

**Supplementary Figure Legends**

**Fig. S1 AJ-5 induces several markers of autophagy in RMS cells.** (A) Representative light microcopy (200X; EVOS XL AMEX1000 Core Imaging System) and maximum intensity projection fluorescence images showing vacuolar structures and acidic vesicles respectively in RH30 and RD cells treated as inducated for 24h. Black arrow heads indicate vacuolar structures. Scale bar is 20 µm. (B) Western blotting showing LC3I and LC3II protein levels in RH30 and RD cells treated with vehicle (V), 0.1 µM or IC_50_ AJ-5 for 24 and 48 h. p38 Was used as a loading control and densitometry readings were obtained using ImageJ. Protein expression levels are represented as a ratio of protein of interest/loading control normalized to a control sample. Blots are representative of at least two independent repeats. (C) Representative maximum intensity projection confocal immunofluorescence images (630X; Zeiss LSM 510; scale bar is 20µm) from three independent repeats of RH30 and RD cells treated with IC_50_ AJ-5 or vehicle for 6, 24 and 48 h and incubated with LC3 primary antibody, fluorophore conjugated Cy3 secondary antibody and nuclei were stained with DAPI.

**Fig. S2 AJ-5 is cytotoxic in a range of sarcoma subtypes.** (A) MTT cell viability assays of chondrosarcoma (SW1353), fibrosarcoma (HT1080), liposarcoma (SW872), osteosarcoma (MG63) and synovial sarcoma (SW982) cells treated with a range of AJ-5 concentrations (0.1 µM – 1.0 µM) or vehicle for 48 h. Graphs show mean cell viability as a percentage of vehicle control ± SEM for each concentration of AJ-5 determined from three independent experiments performed in quadruplicate. A curve was fitted to determine the IC_50_ concentration of AJ-5 for each cell line. (B) Selectivity indices (SIs) were determined for each cell line by dividing the IC_50_ of each non-malignant cell line (Fig. 1C) including normal fibroblast cell lines (FG0 and DMB) and mesenchymal stem cell line (A10021501) by the IC_50_ of each sarcoma cell line. (C) Representative images (top panel) and quantification (lower panel) of clonogenic assays of sarcoma cell lines treated with vehicle, ¼ IC_50_, ½ IC_50_ or IC_50_ concentrations of AJ-5 for 24 h and then replated at low densities in drug-free medium and left for 7-21 days for colonies to form. Colonies were stained with crystal violet and images from three independent repeats were quantified using the ImageJ plugin ColonyArea. The graph represents the mean colony area ± SEM of each treatment condition as a percentage of the vehicle control. Data was analysed using GraphPad Prism 6.0 and a parametric unpaired t-test was performed *p<0.05, **p<0.01, ***p<0.001.

**Fig. S3** Whole blood concentration of AJ-5 over 24 h following a single dose of (A) 2 mg/kg intravenous (IV) (n=3), (B) 2 mg/kg intraperitoneal (IP) (n=3) or 20 mg/kg oral (PO) (n=2) administration of AJ-5 in healthy MF1 mice.

**Table S1** Pharmacokinetic parameters of AJ-5 obtained from whole blood of healthy MF1 mice following a single dose of intravenous (IV), intraperitoneal (IP) or oral (PO) administration of AJ-5 for 24 h. C_max_ = maximum concentration observed; T_max_ = time at maximum concentration observed; t_1/2_ = half-life i.e. time for concentration of drug to decrease by half; CL = drug clearance; Vd = volume of distribution; AUC_0-∞_ = total area under the concentration-time curve i.e. drug exposure; BA = bioavaliability i.e. fraction of administered drug that reached the s; ND = not determined.
