# Supplementary figures and images for: The palladacycle complex AJ-5 induces apoptotic cell death while reducing autophagic flux in rhabdomyosarcoma cells
Source: Cell Death Discov. 2019 Jan 28;5:60. doi: 10.1038/s41420-019-0139-9 (PMC6349869; doi:10.1038/s41420-019-0139-9)

**A**

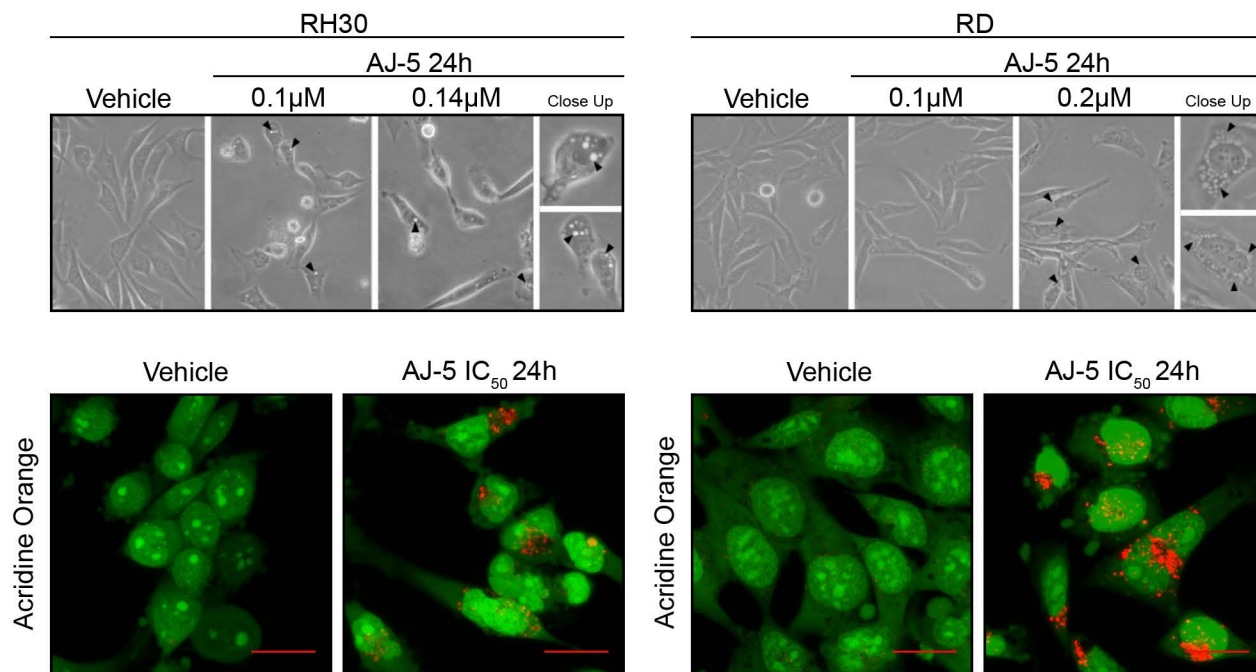

**B**

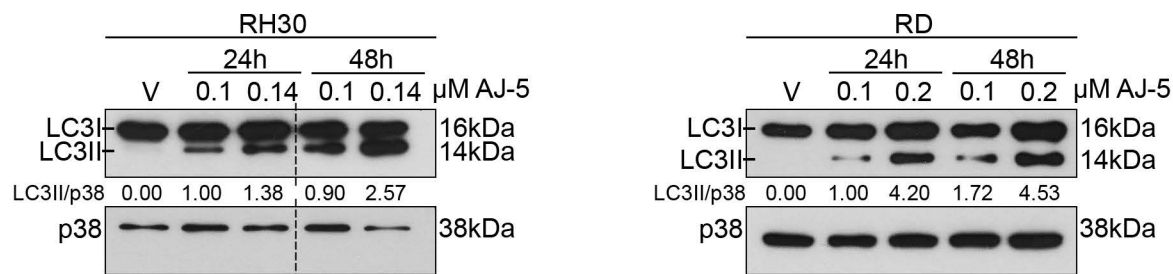

**C**

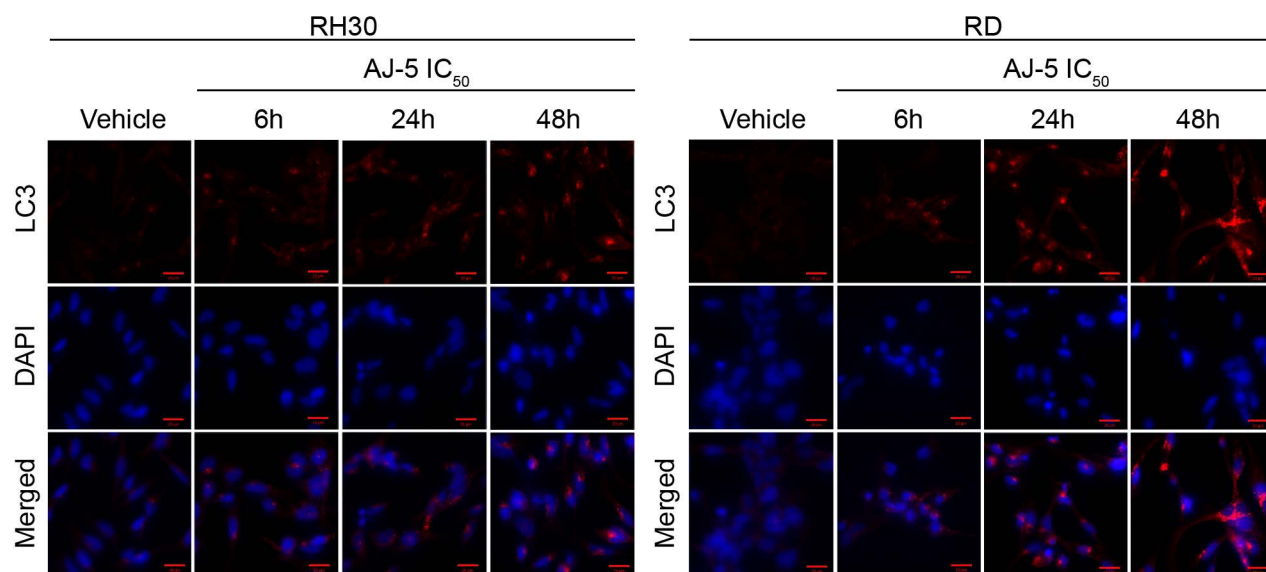

Supplement: Supplementary file 3 — AJ-5 is cytotoxic in a range of sarcoma subtypes [file 41420_2019_139_MOESM3_ESM.pdf]

**A**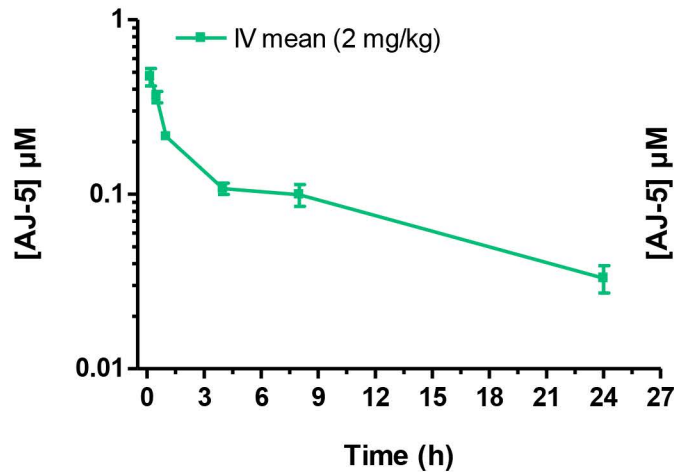**B**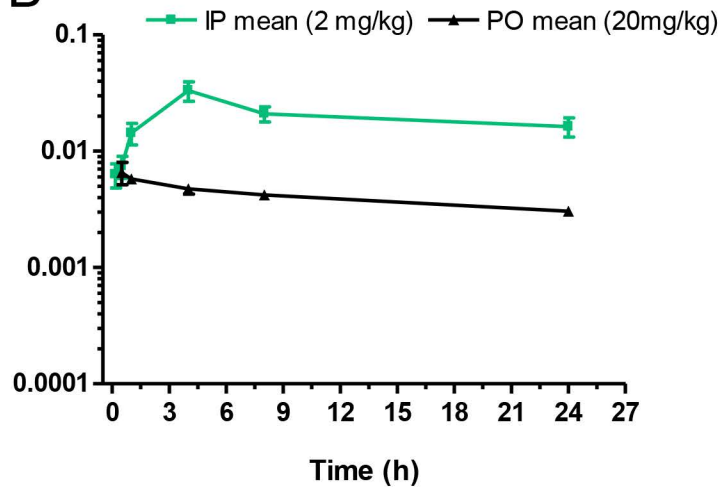

Supplement: Supplementary file 5 — Supplemental Material File #1 [file 41420_2019_139_MOESM5_ESM.pdf]
