# Supplementary material for: The palladacycle complex AJ-5 induces apoptotic cell death while reducing autophagic flux in rhabdomyosarcoma cells
Source: Cell Death Discov. 2019 Jan 28;5:60. doi: 10.1038/s41420-019-0139-9 (PMC6349869; doi:10.1038/s41420-019-0139-9)
Supplement: Supplementary file 4 — Whole blood concentration of AJ-5 over 24h [file 41420_2019_139_MOESM4_ESM.pdf]

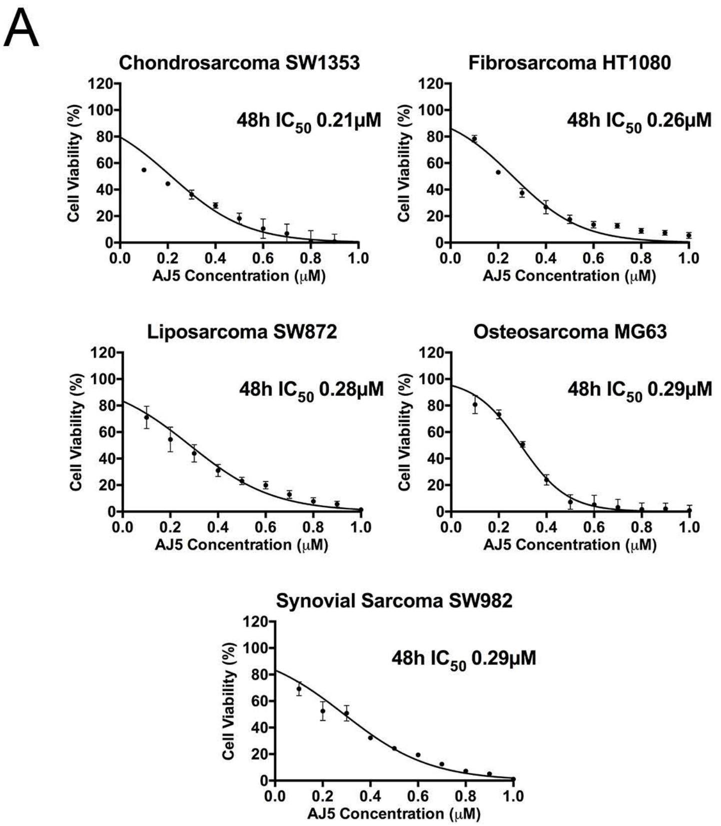

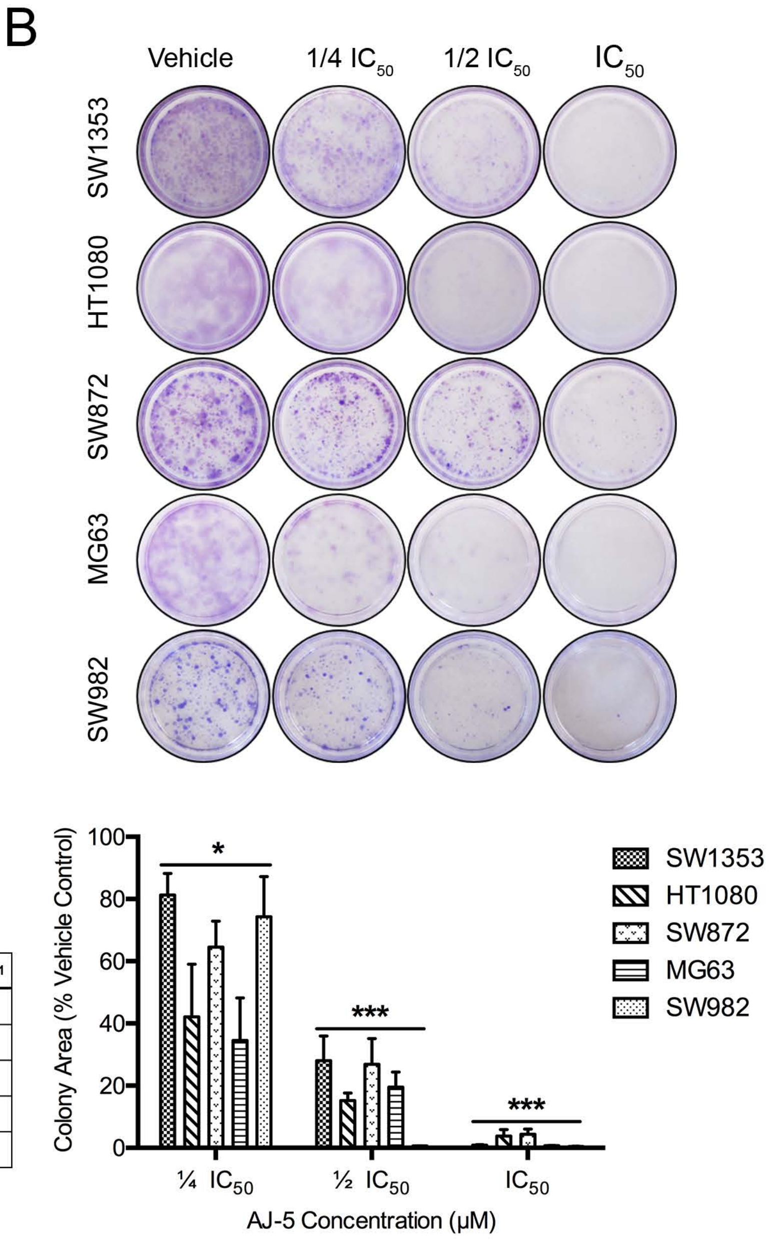

C

$$Selectivity\ Index\ (SI) = \frac{IC_{50}\ Normal\ Cell\ Line}{IC_{50}\ Cancer\ Cell\ Line}$$

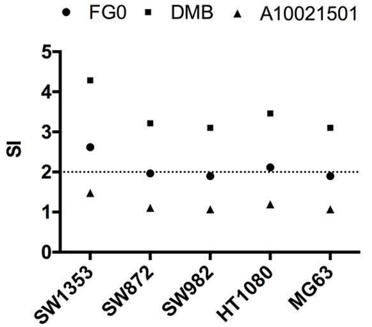

SI Values

	FG0	DMB	A10021501
SW1353	2.62	4.29	1.48
SW872	1.96	3.21	1.11
SW982	1.90	3.10	1.07
HT1080	2.12	3.46	1.19
MG63	1.90	3.10	1.07
